# Supplementary material for: Resolving the full spectrum of human genome variation using Linked-Reads
Source: Genome Res. 2019 Apr;29(4):635–45. doi: 10.1101/gr.234443.118 (PMC6442396; doi:10.1101/gr.234443.118)
Supplement: Supplemental Material [file supp_gr.234443.118_Supplemental_longranger-2.2.zip › longranger-2.2.tar.bz2!/lr2.2-public/lib/stan/cmdstan/stan/doc/api/html/classes.html]

Stan: Class Index


|  |  |
| --- | --- |
|  | Stan  2.5.0  probability, sampling & optimization |


- Main Page
- Related Pages
- Namespaces
- Classes
- Files

- Class List
- Class Index
- Class Hierarchy
- Class Members


All Classes Namespaces Files Functions Variables Typedefs Enumerations Enumerator Friends Macros Pages

Class Index

A | B | C | D | E | F | G | I | J | L | M | N | O | P | R | S | T | U | V | W

|  |  |  |  |  |
| --- | --- | --- | --- | --- |
| B | arg\_stepsize (stan::gm) | function\_signatures (stan::gm) | prob\_grad (stan::model) | seq\_view< T, std::vector< S > > (stan::math) |
|  | arg\_stepsize\_jitter (stan::gm) | functions\_grammar (stan::gm) | program (stan::gm) | seq\_view< T, std::vector< std::vector< T > > > (stan::math) |
| BFGSLineSearch (stan::optimization) | arg\_test (stan::gm) | fvar (stan::agrad) | program\_grammar (stan::gm) | seq\_view< T, std::vector< T > > (stan::math) |
| BFGSMinimizer (stan::optimization) | arg\_test\_grad\_eps (stan::gm) | g | promote\_scalar\_struct (stan::math) | set\_allows\_sampling\_origin (stan::gm) |
| BFGSUpdate\_HInv (stan::optimization) | arg\_test\_grad\_err (stan::gm) |  | promote\_scalar\_struct< T, Eigen::Matrix< S, 1,-1 > > (stan::math) | set\_double\_range\_lower |
| C | arg\_test\_gradient (stan::gm) | general\_matrix\_matrix\_product< Index, stan::agrad::var, LhsStorageOrder, ConjugateLhs, stan::agrad::var, RhsStorageOrder, ConjugateRhs, ColMajor > (Eigen::internal) | promote\_scalar\_struct< T, Eigen::Matrix< S,-1, 1 > > (stan::math) | set\_double\_range\_upper |
|  | arg\_thin (stan::gm) | general\_matrix\_vector\_product< Index, stan::agrad::var, ColMajor, ConjugateLhs, stan::agrad::var, ConjugateRhs > (Eigen::internal) | promote\_scalar\_struct< T, Eigen::Matrix< S,-1,-1 > > (stan::math) | set\_fun\_type (stan::gm) |
| ConvergenceOptions (stan::optimization) | arg\_tolerance (stan::gm) | general\_matrix\_vector\_product< Index, stan::agrad::var, RowMajor, ConjugateLhs, stan::agrad::var, ConjugateRhs > (Eigen::internal) | promote\_scalar\_struct< T, std::vector< S > > (stan::math) | set\_fun\_type2 (stan::gm) |
| D | arg\_unit\_e (stan::gm) | generate\_init\_vars\_visgen (stan::gm) | promote\_scalar\_struct< T, T > (stan::math) | set\_fun\_type3 (stan::gm) |
|  | argument (stan::gm) | generate\_init\_visgen (stan::gm) | promote\_scalar\_type (stan::math) | set\_fun\_type\_named (stan::gm) |
| DoubleVectorView (stan) | argument\_parser (stan::gm) | generate\_local\_var\_init\_nan\_visgen (stan::gm) | promote\_scalar\_type< T, Eigen::Matrix< S, 1, Eigen::Dynamic > > (stan::math) | set\_int\_range\_lower |
| DoubleVectorView< true, false > (stan) | argument\_probe (stan::gm) | i | promote\_scalar\_type< T, Eigen::Matrix< S, Eigen::Dynamic, 1 > > (stan::math) | set\_int\_range\_upper |
| DoubleVectorView< true, true > (stan) | array\_builder (stan::math) |  | promote\_scalar\_type< T, Eigen::Matrix< S, Eigen::Dynamic, Eigen::Dynamic > > (stan::math) | set\_param\_ranges\_visgen (stan::gm) |
| L | array\_literal (stan::gm) | include\_summand (stan::prob) | promote\_scalar\_type< T, std::vector< S > > (stan::math) | set\_var\_type (stan::gm) |
|  | assignment (stan::gm) | increment\_log\_prob\_statement (stan::gm) | promoter (stan::math) | set\_void\_function (stan::gm) |
| LBFGSUpdate (stan::optimization) | b | index\_op (stan::gm) | promoter< Eigen::Matrix< F, R, C >, Eigen::Matrix< T, R, C > > (stan::math) | significant\_decimals\_default\_impl< stan::agrad::var, false > (Eigen::internal) |
| LDLT\_alloc (stan::agrad) |  | index\_type (stan::math) | promoter< Eigen::Matrix< T, R, C >, Eigen::Matrix< T, R, C > > (stan::math) | simplex\_var\_decl (stan::gm) |
| LDLT\_factor (stan::math) | bare\_type\_grammar (stan::gm) | index\_type< const T > (stan::math) | promoter< std::vector< F >, std::vector< T > > (stan::math) | singleton\_argument (stan::gm) |
| LDLT\_factor< stan::agrad::var, R, C > (stan::math) | base\_adaptation (stan::mcmc) | index\_type< Eigen::Matrix< T, R, C > > (stan::math) | promoter< std::vector< T >, std::vector< T > > (stan::math) | size\_of\_helper (stan) |
| LDLT\_factor< T, R, C > (stan::math) | base\_adapter (stan::mcmc) | index\_type< std::vector< T > > (stan::math) | promoter< T, T > (stan::math) | size\_of\_helper< T, true > (stan) |
| LSOptions (stan::optimization) | base\_hamiltonian (stan::mcmc) | init\_local\_var\_visgen (stan::gm) | ps\_point (stan::mcmc) | stack\_alloc (stan::memory) |
| M | base\_hmc (stan::mcmc) | int\_literal (stan::gm) | r | stan\_csv (stan::io) |
|  | base\_integrator (stan::mcmc) | int\_var\_decl (stan::gm) |  | stan\_csv\_adaptation (stan::io) |
| ModelAdaptor (stan::optimization) | base\_leapfrog (stan::mcmc) | integrate\_ode (stan::gm) | range (stan::gm) | stan\_csv\_metadata (stan::io) |
| N | base\_mcmc (stan::mcmc) | is\_constant (stan) | reader (stan::io) | stan\_csv\_reader (stan::io) |
|  | base\_nuts (stan::mcmc) | is\_constant\_struct (stan) | reject\_statement (stan::gm) | stan\_csv\_timing (stan::io) |
| NoOpFunctor (stan::common) | base\_static\_hmc (stan::mcmc) | is\_constant\_struct< Eigen::Block< T > > (stan) | remove\_loop\_identifier (stan::gm) | statement (stan::gm) |
| NumTraits< stan::agrad::var > (Eigen) | base\_var\_decl (stan::gm) | is\_constant\_struct< Eigen::Matrix< T, R, C > > (stan) | unscope\_variables::result (stan::gm) | statement\_2\_grammar (stan::gm) |
| O | binary\_op (stan::gm) | is\_constant\_struct< std::vector< T > > (stan) | binary\_op\_expr::result (stan::gm) | statement\_grammar (stan::gm) |
|  | binary\_op\_expr (stan::gm) | is\_fvar (stan) | add\_conditional\_body::result (stan::gm) | statement\_visgen (stan::gm) |
| OperandsAndPartials (stan::agrad) | bounded (stan::math::detail) | is\_fvar< stan::agrad::fvar< T > > (stan) | set\_double\_range\_upper::result | statements (stan::gm) |
| V | bounded< T\_y, T\_low, T\_high, T\_result, true > (stan::math::detail) | is\_nil\_op (stan::gm) | validate\_int\_data\_expr::result | stepsize\_adaptation (stan::mcmc) |
|  | c | is\_no\_op\_statement\_vis (stan::gm) | add\_expression\_dimss::result (stan::gm) | stepsize\_adapter (stan::mcmc) |
| VectorView (stan) |  | is\_var (stan) | logical\_negate\_expr::result (stan::gm) | stepsize\_covar\_adapter (stan::mcmc) |
| VectorView< const double, false, false > (stan) | categorical\_argument (stan::gm) | is\_var< stan::agrad::var > (stan) | copy\_square\_cholesky\_dimension\_if\_necessary::result | stepsize\_var\_adapter (stan::mcmc) |
| VectorView< const T, is\_array, throw\_if\_accessed > (stan) | chainable (stan::agrad) | is\_var\_or\_arithmetic (stan) | validate\_expr\_type3::result (stan::gm) | store\_type (stan::math) |
| VectorViewMvt (stan) | chainable\_alloc (stan::agrad) | is\_vector (stan) | set\_fun\_type3::result (stan::gm) | store\_type< double > (stan::math) |
| VectorViewMvt< const T, is\_array, throw\_if\_accessed > (stan) | chains (stan::mcmc) | is\_vector< const T > (stan) | addition\_expr3::result (stan::gm) | store\_type< int > (stan::math) |
| a | cholesky\_corr\_var\_decl (stan::gm) | is\_vector< Eigen::Block< T > > (stan) | subtraction\_expr3::result (stan::gm) | stored\_gradient\_vari (stan::agrad) |
|  | cholesky\_factor\_var\_decl (stan::gm) | is\_vector< Eigen::Matrix< T, 1, Eigen::Dynamic > > (stan) | set\_fun\_type2::result (stan::gm) | subtraction\_expr3 (stan::gm) |
| accumulator (stan::math) | cmd\_line (stan::io) | is\_vector< Eigen::Matrix< T, Eigen::Dynamic, 1 > > (stan) | validate\_non\_void\_arg\_function::result (stan::gm) | sum\_values (stan::common::recorder) |
| adapt\_dense\_e\_nuts (stan::mcmc) | common\_type (stan::math) | is\_vector< std::vector< T > > (stan) | set\_void\_function::result (stan::gm) | t |
| adapt\_dense\_e\_static\_hmc (stan::mcmc) | common\_type< Eigen::Matrix< T1, R, C >, Eigen::Matrix< T2, R, C > > (stan::math) | is\_vector\_like (stan) | set\_allows\_sampling\_origin::result (stan::gm) |  |
| adapt\_diag\_e\_nuts (stan::mcmc) | common\_type< std::vector< T1 >, std::vector< T2 > > (stan::math) | is\_vector\_like< const T > (stan) | validate\_declarations::result (stan::gm) | term\_grammar (stan::gm) |
| adapt\_diag\_e\_static\_hmc (stan::mcmc) | conditional\_statement (stan::gm) | is\_vector\_like< Eigen::Matrix< T, Eigen::Dynamic, Eigen::Dynamic > > (stan) | add\_function\_signature::result (stan::gm) | transpose\_expr (stan::gm) |
| adapt\_unit\_e\_nuts (stan::mcmc) | constrained\_param\_names\_visgen (stan::gm) | is\_vector\_like< T \* > (stan) | validate\_return\_type::result (stan::gm) | type\_name (stan::gm) |
| adapt\_unit\_e\_static\_hmc (stan::mcmc) | contains\_fvar (stan) | j | elt\_division\_expr::result (stan::gm) | type\_name< bool > (stan::gm) |
| add\_conditional\_body (stan::gm) | contains\_nonparam\_var (stan::gm) |  | scope\_lp::result (stan::gm) | type\_name< double > (stan::gm) |
| add\_conditional\_condition (stan::gm) | contains\_var (stan::gm) | json\_data (stan::json) | add\_conditional\_condition::result (stan::gm) | type\_name< int > (stan::gm) |
| add\_expression\_dimss (stan::gm) | copy\_square\_cholesky\_dimension\_if\_necessary | json\_data\_handler (stan::json) | set\_int\_range\_lower::result | type\_name< std::string > (stan::gm) |
| add\_fun\_var (stan::gm) | corr\_matrix\_var\_decl (stan::gm) | json\_error (stan::json) | division\_expr::result (stan::gm) | type\_name< unsigned int > (stan::gm) |
| add\_function\_signature (stan::gm) | coupled\_ode\_observer (stan::math) | json\_handler (stan::json) | validate\_return\_allowed::result (stan::gm) | u |
| add\_loop\_identifier (stan::gm) | coupled\_ode\_system (stan::math) | l | validate\_void\_return\_allowed::result (stan::gm) |  |
| add\_var | coupled\_ode\_system< F, double, double > (stan::math) |  | validate\_assignment::result (stan::gm) | unary\_op (stan::gm) |
| add\_while\_body (stan::gm) | coupled\_ode\_system< F, double, stan::agrad::var > (stan::math) | left\_division\_expr (stan::gm) | set\_fun\_type::result (stan::gm) | unconstrained\_param\_names\_visgen (stan::gm) |
| add\_while\_condition (stan::gm) | coupled\_ode\_system< F, stan::agrad::var, double > (stan::math) | list\_argument (stan::gm) | validate\_sample::result (stan::gm) | unit\_e\_metric (stan::mcmc) |
| addition\_expr3 (stan::gm) | coupled\_ode\_system< F, stan::agrad::var, stan::agrad::var > (stan::math) | local\_var\_decl\_visgen (stan::gm) | expression\_as\_statement::result (stan::gm) | unit\_e\_nuts (stan::mcmc) |
| arg\_adapt (stan::gm) | cov\_matrix\_var\_decl (stan::gm) | logical\_negate\_expr (stan::gm) | unscope\_locals::result (stan::gm) | unit\_e\_point (stan::mcmc) |
| arg\_adapt\_delta (stan::gm) | covar\_adaptation (stan::mcmc) | m | add\_while\_condition::result (stan::gm) | unit\_e\_static\_hmc (stan::mcmc) |
| arg\_adapt\_engaged (stan::gm) | csv (stan::common::recorder) |  | add\_while\_body::result (stan::gm) | unit\_vector\_var\_decl (stan::gm) |
| arg\_adapt\_gamma (stan::gm) | csv\_writer (stan::io) | matrix\_var\_decl (stan::gm) | add\_loop\_identifier::result (stan::gm) | unscope\_locals (stan::gm) |
| arg\_adapt\_init\_buffer (stan::gm) | d | mcmc\_writer (stan::io) | remove\_loop\_identifier::result (stan::gm) | unscope\_variables (stan::gm) |
| arg\_adapt\_kappa (stan::gm) |  | member\_var\_decl\_visgen (stan::gm) | validate\_int\_expr2::result (stan::gm) | unvalued\_argument (stan::gm) |
| arg\_adapt\_t0 (stan::gm) | data\_only\_expression | messages (stan::common::recorder) | validate\_allow\_sample::result (stan::gm) | v |
| arg\_adapt\_term\_buffer (stan::gm) | dense\_e\_metric (stan::mcmc) | model\_functional (stan::model) | validate\_non\_void\_expression::result (stan::gm) |  |
| arg\_adapt\_window (stan::gm) | dense\_e\_nuts (stan::mcmc) | modulus\_expr (stan::gm) | validate\_integrate\_ode::result (stan::gm) | validate\_allow\_sample (stan::gm) |
| arg\_bfgs (stan::gm) | dense\_e\_point (stan::mcmc) | multiplication\_expr (stan::gm) | set\_fun\_type\_named::result (stan::gm) | validate\_assignment (stan::gm) |
| arg\_data (stan::gm) | dense\_e\_static\_hmc (stan::mcmc) | n | exponentiation\_expr::result (stan::gm) | validate\_decl\_constraints |
| arg\_data\_file (stan::gm) | diag\_e\_metric (stan::mcmc) |  | multiplication\_expr::result (stan::gm) | validate\_declarations (stan::gm) |
| arg\_decl (stan::gm) | diag\_e\_nuts (stan::mcmc) | name\_vis (stan::gm) | modulus\_expr::result (stan::gm) | validate\_double\_expr |
| arg\_dense\_e (stan::gm) | diag\_e\_point (stan::mcmc) | negate\_expr (stan::gm) | left\_division\_expr::result (stan::gm) | validate\_expr\_type3 (stan::gm) |
| arg\_diag\_e (stan::gm) | diag\_e\_static\_hmc (stan::mcmc) | nil (stan::gm) | elt\_multiplication\_expr::result (stan::gm) | validate\_identifier |
| arg\_diagnose (stan::gm) | distribution (stan::gm) | no\_op (stan::common::recorder) | negate\_expr::result (stan::gm) | validate\_int\_data\_expr |
| arg\_diagnostic\_file (stan::gm) | division\_expr (stan::gm) | no\_op\_statement (stan::gm) | transpose\_expr::result (stan::gm) | validate\_int\_expr |
| arg\_engine (stan::gm) | double\_literal (stan::gm) | numeric\_limits< stan::agrad::fvar< T > > (std) | set\_var\_type::result (stan::gm) | validate\_int\_expr2 (stan::gm) |
| arg\_fail (stan::gm) | double\_var\_decl (stan::gm) | numeric\_limits< stan::agrad::var > (std) | validate\_int\_expr3::result (stan::gm) | validate\_int\_expr3 (stan::gm) |
| arg\_fixed\_param (stan::gm) | dump (stan::io) | nuts\_util (stan::mcmc) | add\_var::result | validate\_integrate\_ode (stan::gm) |
| arg\_history\_size (stan::gm) | dump\_factory (stan::common) | o | validate\_identifier::result | validate\_non\_void\_arg\_function (stan::gm) |
| arg\_hmc (stan::gm) | dump\_member\_var\_visgen (stan::gm) |  | validate\_decl\_constraints::result | validate\_non\_void\_expression (stan::gm) |
| arg\_id (stan::gm) | dump\_reader (stan::io) | op\_ddv\_vari (stan::agrad) | empty\_range::result | validate\_return\_allowed (stan::gm) |
| arg\_init (stan::gm) | dump\_writer (stan::io) | op\_dv\_vari (stan::agrad) | validate\_int\_expr::result | validate\_return\_type (stan::gm) |
| arg\_init\_alpha (stan::gm) | e | op\_dvd\_vari (stan::agrad) | validate\_double\_expr::result | validate\_sample (stan::gm) |
| arg\_int\_time (stan::gm) |  | op\_dvv\_vari (stan::agrad) | set\_double\_range\_lower::result | validate\_transformed\_params\_visgen (stan::gm) |
| arg\_iter (stan::gm) | elt\_division\_expr (stan::gm) | op\_matrix\_vari (stan::agrad) | add\_fun\_var::result (stan::gm) | validate\_var\_decl\_visgen (stan::gm) |
| arg\_lbfgs (stan::gm) | elt\_multiplication\_expr (stan::gm) | op\_v\_vari (stan::agrad) | set\_int\_range\_upper::result | validate\_void\_return\_allowed (stan::gm) |
| arg\_max\_depth (stan::gm) | empty\_range | op\_vd\_vari (stan::agrad) | return\_statement (stan::gm) | value\_type (stan::math) |
| arg\_method (stan::gm) | error\_codes (stan::gm) | op\_vdd\_vari (stan::agrad) | return\_type (stan) | value\_type< const T > (stan::math) |
| arg\_metric (stan::gm) | error\_index (stan) | op\_vdv\_vari (stan::agrad) | returns\_type\_vis (stan::gm) | value\_type< Eigen::Matrix< T, R, C > > (stan::math) |
| arg\_newton (stan::gm) | expl\_leapfrog (stan::mcmc) | op\_vector\_vari (stan::agrad) | row\_vector\_var\_decl (stan::gm) | value\_type< std::vector< T > > (stan::math) |
| arg\_num\_samples (stan::gm) | exponentiation\_expr (stan::gm) | op\_vv\_vari (stan::agrad) | s | valued\_argument (stan::gm) |
| arg\_num\_warmup (stan::gm) | expr\_type (stan::gm) | op\_vvd\_vari (stan::agrad) |  | values (stan::common::recorder) |
| arg\_nuts (stan::gm) | expression (stan::gm) | op\_vvv\_vari (stan::agrad) | sample (stan::gm) | var (stan::agrad) |
| arg\_optimize (stan::gm) | expression07\_grammar (stan::gm) | ordered\_var\_decl (stan::gm) | sample (stan::mcmc) | var\_adaptation (stan::mcmc) |
| arg\_optimize\_algo (stan::gm) | expression\_as\_statement (stan::gm) | p | scalar\_product\_traits< double, stan::agrad::var > (Eigen::internal) | var\_context (stan::io) |
| arg\_output (stan::gm) | expression\_grammar (stan::gm) |  | scalar\_product\_traits< stan::agrad::var, double > (Eigen::internal) | var\_context\_factory (stan::common) |
| arg\_output\_file (stan::gm) | expression\_type\_vis (stan::gm) | partials\_vari (stan::agrad) | scalar\_type (stan) | var\_decl (stan::gm) |
| arg\_random (stan::gm) | expression\_visgen (stan::gm) | pass\_type (stan::math) | scalar\_type< Eigen::Matrix< T, Eigen::Dynamic, Eigen::Dynamic > > (stan) | var\_decls\_grammar (stan::gm) |
| arg\_refresh (stan::gm) | f | pass\_type< double > (stan::math) | scalar\_type< T \* > (stan) | var\_resizing\_visgen (stan::gm) |
| arg\_rwm (stan::gm) |  | pass\_type< int > (stan::math) | scalar\_type\_pre (stan) | var\_size\_validating\_visgen (stan::gm) |
| arg\_sample (stan::gm) | filtered\_values (stan::common::recorder) | positive\_ordered\_var\_decl (stan::gm) | scope\_lp (stan::gm) | vari (stan::agrad) |
| arg\_sample\_algo (stan::gm) | fixed\_param\_sampler (stan::mcmc) | precomp\_v\_vari (stan::agrad) | seq\_view (stan::math) | variable (stan::gm) |
| arg\_save\_iterations (stan::gm) | for\_statement (stan::gm) | precomputed\_gradients\_vari (stan::agrad) | seq\_view< double, std::vector< int > > (stan::math) | variable\_dims (stan::gm) |
| arg\_save\_warmup (stan::gm) | fun (stan::gm) | print\_statement (stan::gm) | seq\_view< T, Eigen::Matrix< S, 1, Eigen::Dynamic > > (stan::math) | variable\_map (stan::gm) |
| arg\_seed (stan::gm) | function\_decl\_def (stan::gm) | printable (stan::gm) | seq\_view< T, Eigen::Matrix< S, Eigen::Dynamic, 1 > > (stan::math) | vector\_var\_decl (stan::gm) |
| arg\_static (stan::gm) | function\_decl\_defs (stan::gm) | printable\_visgen (stan::gm) | seq\_view< T, Eigen::Matrix< S, Eigen::Dynamic, Eigen::Dynamic > > (stan::math) | visgen (stan::gm) |
|  |  |  |  |  |

A | B | C | D | E | F | G | I | J | L | M | N | O | P | R | S | T | U | V | W


---

[ Stan Home Page ]

*© 2011–2014,
Stan Development Team.*
